# General Anaesthesia Versus Conscious Sedation in Transcatheter Aortic Valve Implantation: Differences in Pulmonary Infections

**DOI:** 10.31083/RCM48924

**Published:** 2026-05-26

**Authors:** Astrid Bergmann, Helmut Warkentin, Florian Hildebrandt, Janis Fliegenschmidt, Nikolai Hulde, Tanja Rudolph, Smita Scholtz, Cornelia Piper, Sabine Bleiziffer, Vera von Dossow

**Affiliations:** ^1^Ruhr-University Bochum, Heart- and Diabetes-Center, 32545 Bad Oeynhausen, Germany; ^2^University Bielefeld, Medical Center East Westphalia-Lippe, Heart- and Diabetes-Center, 32545 Bad Oeynhausen, Germany; ^3^Telehealth Competence Center (TCC), 22083 Hamburg, Germany; ^4^Department of Cardiac Surgery, Zentralklinik Bad Berka, Rhön Klinikum AG, 99438 Bad Berka, Germany

**Keywords:** transcatheter aortic valve replacement, general anaesthesia, conscious sedation, pneumonia, propensity score matching, renal replacement therapy

## Abstract

**Background:**

Patients undergoing transfemoral aortic valve replacement are particularly vulnerable and require a more sophisticated anesthetic therapeutic approach. According to the literature, no study has directly compared general anaesthesia with conscious analgosedation using postoperative infections as the primary endpoint.

**Methods:**

Patients undergoing transcatheter aortic valve implantation (TAVI) were analyzed retrospectively. A total of 3313 patients from a large heart center in Western Europe were included in this study. One group received general anaesthesia, and the other group received analgosedation for TAVI. The primary outcome was postinterventional pneumonia; secondary outcomes included myocardial infarction, renal failure, stroke, and 30-day mortality. Propensity score matching using 16 matching criteria yielded over 1000 pairs.

**Results:**

No difference was observed in the incidence of postinterventional pneumonia (*p* = 0.148). The occurrence of myocardial infarction (*p* = 0.2) and stroke (*p* = 0.4) also did not differ significantly between the two groups. In contrast, the need for transient renal replacement therapy (*p* = 0.02) and 30-day mortality (*p* = 0.02) were lower in the analgosedation group.

**Conclusions:**

Regarding postinterventional pneumonia, general anaesthesia can be used as safely as analgosedation during TAVI. However, since renal failure requiring temporary replacement therapy and mortality are both increased with general anaesthesia, analgosedation should be the standard of care for TAVI in high-volume centers. The anesthetic regimen must be determined on an individual basis and discussed during the heart team briefing. The conversion to, or primary use of, general anaesthesia when clinically indicated is safe. Overall, ensuring the continuous presence of a senior consultant anesthetist, specifically trained in cardiac anaesthesia, throughout the procedure is essential.

## 1. Introduction

Transcatheter-assisted interventional heart valve implantation represents one of the most significant developments in cardiac medicine over the past 15 years [1]. Meanwhile, this technique is characterized by close interdisciplinary collaboration within the heart team, which consists of cardiologists, cardiac surgeons, and cardiac anesthesiologists [2, 3]. Transfemoral aortic valve replacement, a minimally invasive form of aortic valve replacement, is the most common catheter-assisted valve intervention in Germany; meanwhile, the number of cases continues to increase annually [4].

Demographic trends in Germany show that approximately 21% of the population is 65 years of age or older [5]. Consequently, cardiac surgery and interventional procedures are increasingly performed in patients aged 70 years and older. Given the numerous comorbidities and increasing frailty of these individuals, these patients have an increased risk of postoperative complications [6, 7, 8] following surgical aortic valve replacement via sternotomy. Transcatheter aortic valve implantation (TAVI) is now the most commonly performed valve intervention [3, 9, 10] and has taken precedence over surgery in the clinical management of aortic valve stenosis in Germany.

The choice of an anaesthesia technique is a patient-individualized decision, which is discussed with the patient in advance, based on careful information and risk stratification. This involves more than simply choosing between general anaesthesia and analgosedation. Surgical indications are increasingly being made for patients in older age groups, reflecting recent medical–technological developments. Therefore, anesthetic techniques are becoming increasingly more clinically relevant and require a critical examination of the physiological and pathophysiological characteristics of the aging population. For example, older patients are significantly more sensitive to the respiratory depression effects of sedatives and opioids, which increases the risk of postoperative narcotic overload [11, 12]. Reduced esophageal motility can also increase the risk of aspiration in aging patients [13]. During analgosedation, airway protection is not reliably ensured, and the risk of microaspiration with subsequent pulmonary complications is increased. Regarding the circulatory system, older patients also have reduced tolerance to volume status, including fluid depletion or overload [14]. This leads to an increased risk of hemodynamic instability requiring drug-based circulatory therapy. The reduction in renal mass also reduces drug clearance [15], requiring close dosage adjustment.

There has been increasing discussion among representatives of international anaesthesia societies regarding the preferred anesthetic technique for TAVI. Increasing experience with the TAVI procedure and advances in device technology have led to a decline in the use of general anaesthesia during TAVI [16]. In Europe, a trend toward analgosedation, or “light sedation” combined with local anaesthesia, has become established in most major TAVI centers. In the randomized TAVI-SOLVE trial [17], the authors demonstrated comparable results for the analgosedation concept concerning rates of hospital mortality, stroke, and myocardial infarction.

Nonetheless, despite numerous retrospective studies on TAVI procedures, no study has directly compared general anaesthesia with analgosedation using postoperative infections as the primary endpoint. Thus, this study used a retrospective anaesthesia dataset from a single center between 2017 and 2021and compared two in-house anaesthesia procedures, which were stored as treatment pathways (“standard operating procedures”). The primary objective was to examine the effects on postoperative infections, particularly pneumonia. Secondary outcomes included 30-day all-cause mortality, stroke, myocardial infarction, and acute kidney injury. Findings indicating the superiority of one of the two techniques could help optimize patient care and inform recommendations for anesthetic management in patients undergoing TAVI. Furthermore, this study underscores the importance of the continuous presence and support of a consultant anesthetist at the highest professional level during TAVI.

## 2. Materials and Methods

A retrospective, single-center analysis of 3313 patients who underwent transfemoral TAVI between 01/01/2017 and 31/10/2021 was performed. Data were retrieved from the anesthetic records stored in the electronic chart (“Copra-Anästhesie”) of a high-volume cardiac center, as well as from the intensive care unit records from the same institution (“Copra-Intensiv”). All data, including demographic and personal information, were accessible to the researchers conducting retrospective analyses of the records. The primary endpoint, pneumonia, was assessed using a non-inferiority approach (margin of 5% and a significance level of 0.05). Secondary endpoints included 30-day all-cause mortality, stroke, myocardial infarction, and acute kidney injury.

To address potential patient selection bias, propensity score matching based on 16 matching criteria was applied.

The institutional ethics committee approved the study protocol on 18/03/2021 (OWL 2021-778).

### 2.1 Subjects

The COPRA database was analyzed retrospectively; 3313 patients who underwent elective transfemoral TAVI between 01/01/2017 and 31/10/2021 were included in this study. All included patients provided written informed consent for anaesthesia and the TAVI procedure. All patients were permitted to consume clear fluids until two hours before the procedure, and solid food until six hours beforehand. No sedating premedication was administered.

### 2.2 Instrumentation

In the induction room, patients were placed on a heating blanket (Twinwarm® BB, Moeck & Moeck GmbH, Hamburg, Germany) and equipped with standard monitoring equipment consisting of 5-lead-electrocardiogram (Philips IntelliVue patient monitoring system, Philips Medizin Systeme GmbH, Böblingen, Germany), oxygen saturation clip (M1191B, Philips Medizin Systeme GmbH, Böblingen, Germany) on the right index finger, arterial cannula (Arrow® arterial catheterization set, Teleflex Incorporated, Wayne, PA, USA) in the left radial artery for continuous measurement of the arterial pressure and a peripheral venous access (Vasofix® Safety, B.Braun Melsungen AG, Melsungen, Germany) in the lower arm, either on the right or on the left side for the infusion of volume, induction medication and continuous infusion of the anesthetic drugs throughout the procedure. After induction of general anaesthesia or when analgosedation was achieved, a central venous catheter was inserted under ultrasound guidance into the right internal jugular vein for administration of vasoactive drugs. A sheath for intracardiac pacemaker insertion (Arrow® Percutaneous Sheath Introducer Set, Teleflex Incorporated, Wayne, PA, USA) was also placed in the same vessel under ultrasound guidance. During central venous catheter (Certofix®, B. Braun Melsungen AG, Melsungen, Germany) placement, 2 g of cefazolin (Fresenius Kabi Deutschland GmbH, Bad Homburg, Germany) was administered intravenously as antibiotic prophylaxis. In patients who were sedated but not under general anaesthesia, a local anesthetic was injected subcutaneously at the insertion site to provide analgesia before the procedure. A urinary catheter was then inserted. Patients were subsequently transferred to the operating room and positioned on the catheterization table. All syringe pumps were adjusted to ensure the correct delivery of the various medications. Patients were securely positioned to prevent pressure damage, and the heating blanket was activated to maintain a stable body temperature. The depth of anaesthesia or analgosedation was measured using a single-channel electroencephalogram (Narcotrend Compact M, MonitorTechnik GmbH & Co. KG, Bad Bramstedt, Germany) recorded from both hemispheres; the depth of anaesthesia/analgosedation was maintained within the manufacturer’s recommended range.

### 2.3 General Anaesthesia

After adequate preoxygenation with an FiO_2_ of 1, general anaesthesia was induced intravenously with sufentanil (0.05–0.1 µg/kg, Janssen-Cilag GmbH, Neuss, Germany), etomidate (0.2–0.3 mg/kg, B.Braun Melsungen AG, Melsungen, Germany), and rocuronium (0.6–0.9 mg/kg, Organon / MSD, OSS, Netherlands). After the onset of muscle relaxation, an endotracheal tube was inserted under direct laryngoscopy and secured with adhesive tape. General anaesthesia was maintained through the continuous application of propofol (3–4 mg/kg/h, B. Braun Melsungen AG, Melsungen, Germany) and remifentanil (0.3 mg/kg/h, GlaxoSmithKline / Aspen, Brentford, UK).

### 2.4 Analgosedation

Patients were encouraged to breathe four to six liters of oxygen via a facemask, and respiration was monitored by capnography. Sufentanil (5–10 µg, Janssen-Cilag GmbH, Neuss, Germany) was administered intravenously, and continuous infusions of propofol (2–3 mg/kg/h) and dexmedetomidine (0.8–1 µg/kg/h, Orion Pharma, Espoo, Finland) were initiated and maintained throughout the procedure.

### 2.5 Transcatheter Aortic Valve Implantation

The transfemoral aortic valve was implanted in accordance with the cardiological and cardiosurgical standard operating procedures at our institution [18, 19]. Self-expanding and balloon-expandable valves were used in the patients included in the present study. A dose of 150 to 200 IU/kg of unfractionated heparin (B.Braun Melsungen AG, Melsungen, Germany) was administered intravenously after puncture of the femoral artery to achieve an activated clotting time (ACT) of at least 270 seconds. Heparin was antagonized with 100–150 IU/kg of protamine (LEO Pharma A/S, Ballerup, Denmark) at the end of the procedure, when the sheaths were withdrawn.

### 2.6 After TAVI

Once the valve was correctly positioned, as assessed by transthoracic echocardiography and angiography, the anesthetic agents and/or analgosedation were discontinued, and patients were encouraged to wake up. Those patients who had received general anaesthesia were extubated in the operating room and provided oxygen via a face mask at 4–6 L/min. Patients in both groups were then transferred to the intensive care unit for hemodynamic and respiratory monitoring.

### 2.7 Pneumonia

To diagnose pneumonia, key infection parameters were measured. Pneumonia onset was defined as a C-reactive protein (CRP) level >0.5 mg/dL, coupled with clinical signs of infection (cough, fever, or tachypnea) and evidence of infiltrates or consolidation on chest X-ray, warranting antibiotic treatment.

### 2.8 Statistical Analysis

Given the large sample size (n = 3313), formal tests of normality were not used, as these tests tend to be overly sensitive in large samples. Instead, distributional characteristics were assessed visually; variables with approximately symmetric distributions are summarized as the mean (standard deviation, SD). In contrast, variables with clearly skewed distributions or outliers are reported as medians (interquartile ranges, IQRs). To account for differences in baseline characteristics between patients treated with general anaesthesia or analgosedation, 1:1 propensity score matching was performed. Propensity scores were estimated using logistic regression, including the following variables: age, sex, body mass index, preoperative aortic valve assessment, coronary artery disease, EUROSCORE, history of previous cardiac surgery, history of myocardial infarction, diabetes mellitus, chronic obstructive pulmonary disease, neurological issues, renal failure, dialysis, arterial hypertonia, peripheral vascular disease, and previous pacemaker or internal cardioverter defibrillator implantation. Patients were matched in a 1:1 ratio using nearest-neighbor matching without replacement based on the estimated propensity scores. No caliper was applied. Balance between matched groups was assessed by descriptive comparison of baseline characteristics. All statistical analyses were performed using R version 4.4.3 (R Foundation for Statistical Computing, Vienna, Austria) through RStudio version 2024.12.1.563 (Posit PBC, Boston, MA, USA). Propensity score matching was performed using the MatchIt package, version 4.7.2 (open-source R package distributed via CRAN).

## 3. Results

A total of 3313 patients who underwent transfemoral TAVI at our center between 01/01/2017 and 31/10/2021 were retrospectively analyzed. Anesthetic records during the TAVI procedure indicated that 1056 patients received general anaesthesia, whereas 2257 were managed using analgosedation and breathed spontaneously during the procedure. Of the 2257 patients who underwent analgosedation, 1051 were successfully matched to patients who received general anaesthesia. The remaining 1206 patients in the analgosedation group could not be adequately matched to counterparts in the general anaesthesia group and were, therefore, excluded from the matched cohort to avoid biased estimates. After propensity score matching, 1051 pairs remained, yielding a total of 2102 patients.

Demographic data after propensity score matching are presented in Table 1.

**Table 1.** **Demographical data of the study cohort**.

	Overall	GA	AS	
Variable	2102	1051	1051	*p*-value
Sex (male)	914 (43%)	456 (43%)	458 (44%)	0.9
Age	82 (±6)	82 (±6)	82 (±6)	0.4
BMI	27 (±5)	27 (±5)	26 (±5)	0.6
AoS II	182 (9%)	92 (9%)	90 (9%)	0.9
AoS III	1906 (91%)	951 (90%)	955 (91%)	0.9
AR II	82 (4%)	45 (4%)	37 (4%)	0.9
AR III	7 (0.3%)	3 (0.2%)	4 (0.4%)	0.9
CVD 0	907 (43%)	459 (44%)	448 (43%)	0.9
CVD 1	411 (20%)	203 (19%)	208 (20%)	0.9
CVD 2	322 (15%)	157 (15%)	165 (16%)	0.9
CVD 3	462 (22%)	232 (22%)	230 (22%)	0.9
EUROSCORE 2	6 [1; 82]	6 [1; 82]	6 [1; 61]	0.1
MI ≤3 months	56 (3%)	29 (3%)	27 (3%)	0.9
Diabetes mellitus	672 (32%)	336 (32%)	336 (32%)	0.9
COPD	422 (20%)	213 (20%)	209 (20%)	0.9
PAVD	203 (10%)	102 (10%)	101 (9%)	0.9
Arterial hypertonus	1889 (90%)	945 (90%)	944 (90%)	0.7
GFR	56 [5; 121]	56 [5; 111]	57 [5; 121]	0.8
LVEF	52 (±10)	51 (±10)	52 (±9)	0.5

Age, BMI, and LVEF are presented as the mean (± standard deviation (SD)), EUROSCORE 2 and GFR as median [Q1; Q3], and other values as the total percentage of the group. GA, general anaesthesia; AS, analgosedation; BMI, body mass index; AoS, aortic stenosis; AR, aortic regurgitation; CVD, cardiovascular disease; MI, myocardial infarction; COPD, chronic obstructive pulmonary disease; PAVD, peripheral arterial vascular disease; GFR, glomerular filtration rate; LVEF, left ventricular ejection fraction.

**Fig. 1.**
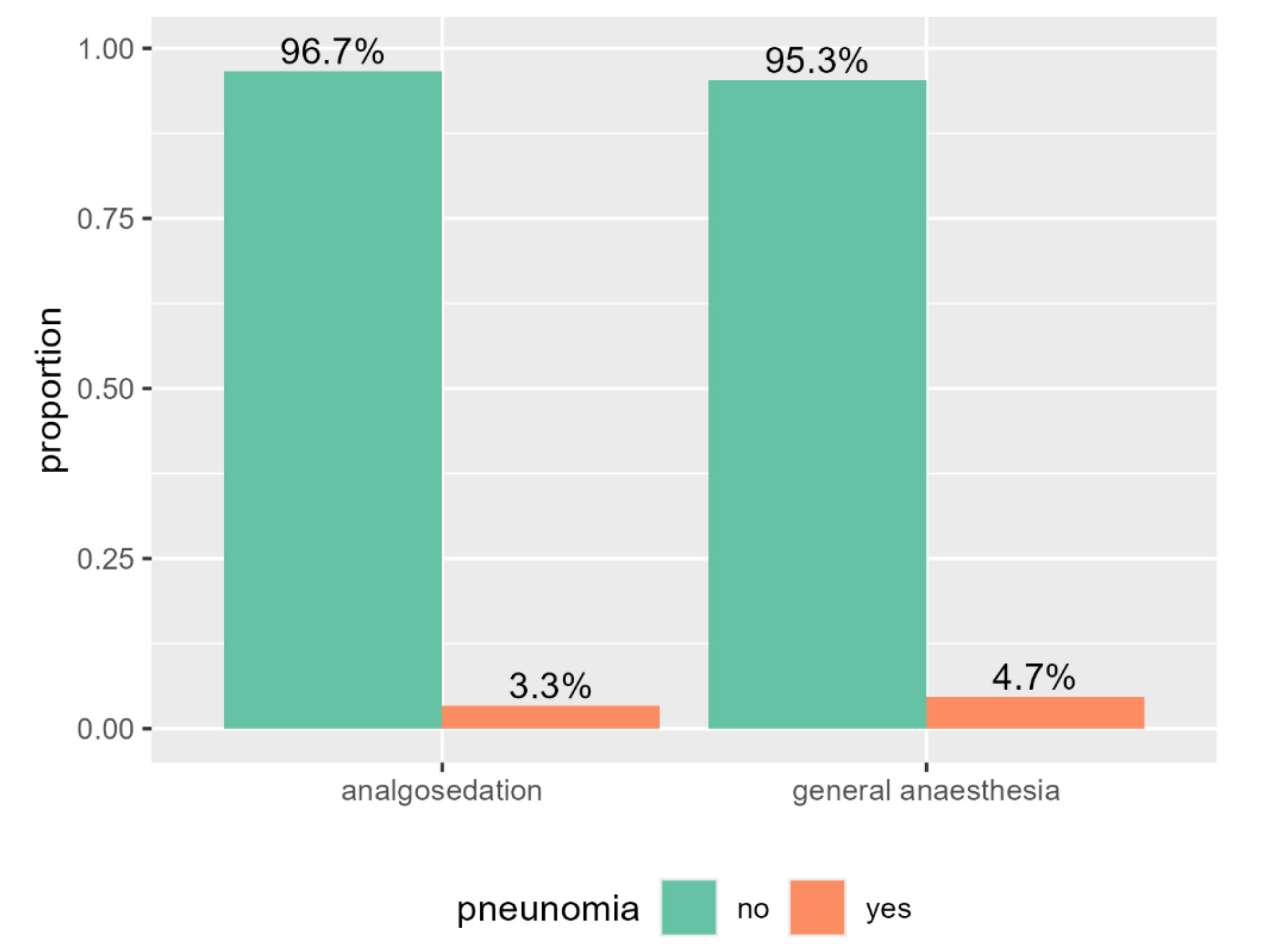
**Bar plot of the study cohort in both groups (analgosedation versus general anaesthesia) and the proportion of pneumonia**.

No significant difference (*p* = 0.148) was detected in the primary outcome, the onset of postoperative/postinterventional pneumonia (Fig. 1).

There were no significant differences between the two groups in the incidence of stroke (*p *= 0.4) or myocardial infarction (*p *= 0.2) within 72 hours after TAVI. The 30-day mortality post-intervention was significantly lower in patients who underwent TAVI under analgosedation (*p *= 0.02), and the incidence of acute kidney injury requiring temporary replacement therapy was also significantly lower in this group (*p *= 0.02). The anesthetic strategy was further associated with significant differences in intensive care unit (ICU) and hospital length of stay (LOS), with a shorter median LOS observed in the analgosedation group compared with the general anaesthesia group. The data are presented in Table 2.

**Table 2.** **Secondary outcome variables**.

	Overall	GA	AS	
Variable	2102	1051	1051	*p*-value
Mortality (30 d)	63 (3.0%)	41 (3.9%)	22 (2.1%)	0.020
Stroke	53 (2.5%)	26 (2.5%)	27 (2.5%)	0.400
MI ≤72 h	3 (0.1%)	0	3 (0.3%)	0.200
RRT (transient)	42 (2.0%)	31 (2.9%)	11 (1.0%)	0.020
ICU LOS (days)	2 [1; 3]	2 [1; 3]	1 [1; 2]	0.001
Hospital LOS (days)	11 [8; 15]	13 [9; 16]	10 [8; 14]	0.001

ICU LOS and hospital LOS are presented as the median [Q1; Q3], other values are reported as totals (percentage of the group). RRT, renal replacement therapy; ICU, intensive care unit; LOS, length of stay.

## 4. Discussion

The main results of this study are that (I) the use of analgosedation instead of general anaesthesia for TAVI is not associated with an increased risk of postinterventional pneumonia; (II) the transient need for renal replacement therapy and 30-day mortality are reduced by analgosedation; (III) the incidence of stroke and myocardial infarction within 72 hours after TAVI is not affected by the use of analgosedation versus general anaesthesia; (IV) ICU and hospital LOS are reduced in the analgosedation group.

These data add to the existing literature by suggesting that analgosedation during TAVI is not associated with an increased risk of postinterventional pneumonia. To our knowledge, this association has not previously been demonstrated in a large propensity score-matched cohort.

We were also unable to confirm previous findings that the risk of adverse events such as stroke and myocardial infarction is not increased with analgosedation for TAVI [20]. A five-year follow-up of the SOLVE-TAVI trial likewise showed no differences in outcomes based on the choice of anesthetic regime [21].

Acute renal failure requiring replacement therapy was actually reduced after analgosedation. Previous studies [22, 23] have shown that the risk of developing acute kidney injury after TAVI is high. This increased risk is due to pre-existing medical conditions that cannot be altered and issues inherent to the procedure, such as the use of contrast agents, rapid pacing, and manipulation of the vascular system. The lower incidence of renal replacement therapy observed in patients receiving analgosedation may be related to differences in intraoperative haemodynamics. One possible explanation is the preservation of systemic vascular resistance and mean arterial pressure compared with general anaesthesia, which may support renal perfusion.

Thirty-day mortality was lower in the analgosedation group. Patients undergoing TAVI are older and usually have multiple comorbidities. Given the retrospective nature of the study and the lack of data on causes of death, no conclusions regarding causality can be drawn.

Aslan *et al*. [24] conducted a study comparing analgosedation and general anaesthesia for TAVI in patients with severe chronic obstructive pulmonary disease (COPD) and concluded that general anaesthesia is associated with increased pulmonary complications after TAVI. The authors recommend the use of analgosedation in patients with chronic pulmonary diseases. In our cohort, both analgosedation and general anaesthesia appeared safe; however, we did not analyze patients with pre-existing lung disease. This question should be addressed in future research.

There may be concerns that the use of analgosedation could lead to respiratory depression, especially in vulnerable and frail patients undergoing TAVI. Our results indicate that analgosedation can be safely used in the TAVI setting with respect to the postinterventional onset of pneumonia. Moreover, oxygenation can be improved with high-flow nasal oxygen therapy during the procedure, as shown by Giménez-Milà *et al*. [25].

Our findings can be reassuring in the provision of anaesthesia during TAVI. During TAVI, it might be necessary to convert from analgosedation to general anaesthesia, *i*.*e*., when the cardiologist requires transesophageal echocardiography for valve implantation or when a change in access route is needed because the percutaneous approach is impeded. General anaesthesia may also be required when patients are unable to lie flat, such as in cases of severe lower back issues or intrinsic agitation. Based on the present findings, analgosedation appears to be a safe and feasible anesthetic approach for TAVI in a high-volume center, with outcomes comparable to general anaesthesia regarding pulmonary and neurological complications. General anaesthesia remains an important option in selected cases and can be applied safely when clinically indicated. We demonstrated that there is no increased risk of pneumonia when conversion to general anaesthesia is required or even when general anaesthesia is used at the start of the procedure. Short-acting agents should be preferred to facilitate on-table extubation without prolonged mechanical ventilation or transfer to the intensive care unit. The depth of anaesthesia should be monitored to avoid oversedation and the need for prolonged hemodynamic monitoring, which may be measured non-invasively.

We emphasize the importance of maintaining the continuous presence of a senior consultant anesthetist, specifically trained in cardiac anaesthesia, to ensure patient safety and comfort. The anesthetic regimen should be determined individually and presented during the heart team briefing, which must include a cardiac anesthetist. This approach ensures the best possible care for each patient, taking into account the individual physical and physiological condition of each patient. It has been demonstrated that the anesthetic regimen affects patient outcomes [26]. Therefore, decisions regarding the regimen are best left to a trained and experienced anesthetist who is present throughout the process and is familiar with both analgosedation and general anaesthesia. The presence of the anesthetist is crucial because complications such as bleeding and major vascular complications, including rupture of the aortic root or ventricle, leading to tamponade and circulatory depression, require immediate recognition and intervention.

General anaesthesia is not without risk, especially in older patients, due to decreased drug clearance, reduced distribution space, drug interactions, cardiovascular depression, mechanical ventilation, and the potential need for inotropic and circulatory support. However, when clinically indicated in the situations described above, general anaesthesia can be administered safely. However, we recommend analgosedation as the standard anesthetic approach during TAVI.

In the present study, anesthetic strategy was associated with significant differences in ICU and hospital length of stay among patients undergoing TAVI. Patients managed with analgosedation had a shorter median LOS than those receiving general anaesthesia. This observation may be explained by the avoidance of prolonged mechanical ventilation or reduced hemodynamic instability. Even modest reductions in ICU stay may be clinically meaningful in high-volume centers, where resource utilization is a critical consideration. However, patients selected for general anaesthesia may have had greater procedural complexity or comorbidity burden. Therefore, the observed association between anesthetic technique and LOS should be interpreted cautiously.

### Limitations

Our study is retrospective and based on a single-center population, which may limit the generalizability of the findings. In addition, potential confounders that may contribute to postinterventional pneumonia, such as hiatal hernia and certain medications, including antidiabetic agents and immunosuppressants, were not excluded from our cohort and warrant further study. Moreover, although baseline characteristics after propensity score-matching were carefully compared, standardized mean differences were not calculated, which represents an additional limitation of the present analysis.

The study period (2017–2021) coincided with a period of continuous evolution in TAVI techniques and increasing operator and heart team experience, particularly regarding conscious sedation. Although these developments affected all patients during the study period, the influence of these issues may not be fully captured by the available variables. Therefore, incremental improvements in procedural execution, patient selection, and peri-procedural management, as well as unmeasured aspects of the learning curve, may have contributed to residual confounding.

Our center is highly specialized in cardiac surgery and in cardiac interventions, which, in turn, might have led to a liberal approach to analgosedation instead of general anaesthesia. Nevertheless, a large number of patients were included in our study cohort, yielding highly relevant results that support the use of analgosedation for TAVI and underscore the need for the obligatory presence of the anesthetist in this setting.

## 5. Conclusions

In this retrospective, single-center analysis, general anaesthesia was not associated with a higher incidence of postinterventional pneumonia compared with analgosedation during TAVI. Analgosedation was associated with lower rates of temporary renal replacement therapy and lower 30-day mortality, while rates of stroke and myocardial infarction were similar between groups.

While causal inferences cannot be drawn, these findings suggest that analgosedation is a safe and potentially advantageous anesthetic strategy for TAVI in experienced centers. The choice of anesthetic regimen should remain individualized and determined by the heart team, with the continuous presence of an experienced cardiac anesthetist.

## Data Availability

The data that support the findings of this study are available from the corresponding author, AB, upon reasonable request.

## Abbreviations

TAVI, transcatheter aortic valve implantation.
